# The Tortoise and the Hut

**DOI:** 10.3201/eid1707.AC1707

**Published:** 2011-07

**Authors:** Polyxeni Potter

**Affiliations:** Author affiliation: Centers for Disease Control and Prevention, Atlanta, Georgia, USA

**Keywords:** art science connection, emerging infectious diseases, art and medicine, Salum Kambi, The Village Hut, the tortoise and the hut, tropical diseases, vector-borne infections, Tanzania, about the cover

“The men of old were born like the wild beasts. In woods, caves, and groves, they lived on food gathered in the fields,” wrote Marcus Vitruvius Pollio more than 2,000 years ago in his well-known account of classical traditions in architecture. One day, a dense group of trees, agitated by winds and storms, caught fire. Humans nearby ran from the flames but then, attracted to the warmth, began to draw near. They tried to keep the flames going and brought others to see them. As they shared the discovery, they gestured, made sounds with their voices, and started to articulate words. Society and language had begun.

“As they kept coming together in greater numbers into one place, they began… to construct shelters” dug in on the sides of mountains, roofed with boughs, or made of twigs and mud like bird nests. They observed each other’s efforts and made improvements. “At first they wove their walls with upright forked props and drove twigs between them…. When in winter-time the huts could not withstand the rains, they made their roofs sloping and projected and, smeared with clay, the ridged roofs drew off the rain water.”

Such were the origins of architecture, rooted in human needs and natural surroundings: a pitched roof made of living materials shaped to more or less resemble a turtle. And apart from its obvious economy and efficiency, the dwelling has endured as a symbol of ecologic equilibrium, made to conform to nature and fit in with the landscape. From the construction of huts, humans progressed to other arts and sciences. With time, harmonious alignment of dwelling with nature and form with function has not always kept pace. Yet, spatial relationships, the way forms interact with those who use them and their surroundings, have remained a main consideration not only in architecture but all the arts and no less so in painting. This consideration as it pertains to color is very prominent in the work of East African painter Salum Kambi, whose work *The Village Hut* graces this month’s issue.

Kambi was born in Dar es Salaam, Tanzania, a culturally diverse nation with more than 120 ethnic groups and rich artistic traditions going back to prehistoric times. A talented youth, he sought opportunities everywhere. “I entered a competition related to research on chimpanzees in the Gombe Forest in Kigoma and was one of five artists selected as the winners and had the chance to meet former President Mwalimu Julius Nyerere.” Kambi apprenticed with artist Mohamed Raza and his son, “They taught me a lot about art and painting.” He worked with cartoonist Godfrey Mwampemba known as Gado. “Having no formal art training, I acquired my skills with the help of these fellow Tanzanian artists and while attending workshops in Dar es Salaam.” For a time, he practiced his trade in Kampala, Uganda, but returned to Tanzania where he had several exhibits. He has also exhibited in the Netherlands, Germany, Finland, Sweden, Italy, Denmark, and the United States.

“For me, art is all about color. I use color to depict feelings… of people, landscapes, animals.” Like the anonymous ancestors of the Sandawe people in central Tanzania, who painted or engraved images on rocks, Kambi depends entirely on color. They used predominantly a single primary color, relying exclusively on red, black, or white values. He uses oil and acrylic paints applied with a knife, a brush, or both to create striking abstractions that exude feeling. In more modern terms, his work recalls the fauvists, the French avant-garde movement of the early 20th century, and their spontaneous expressions in potent color used directly from the tube.

“Fauve art isn’t everything, but it is the foundation of everything,” Henri Matisse believed. “When I put a green, it is not grass. When I put a blue, it is not the sky.” Even if the subject is traditional―a human body, a piece of fruit, a mountain―fauve colors make it look outworldly and unfinished. Form does not determine color. Color is there to create a sensation, so it can be nonrepresentational and unnatural. Choice of color is not right or wrong but the subjective vision of the artist.

*The Village Hut* approaches a primeval subject, shelter, along these unconventional lines. The artist’s palette transforms the ordinary with unexpected hues. He is not interested in likeness so much as intensity or the sky would not be red, the trees not black, the thatched roof not icy white. Constructed of cubes and strokes of color and framed by auxiliary structures, the compound rests comfortably against the brilliant backdrop and the slick, luminescent pathway. This dwelling seems inhabited, vibrant. This hut is a place.

More than 80% of the population of Tanzania is rural. Kambi’s emblematic depiction of standard quarters in the countryside captures not just the dwelling itself with its tortoiselike functionality and simplicity but also its explosive milieu, rich with organic elements of the tropics capable of engulfing the makeshift structure at will. And while for architectural purposes the tortoise analogy works, in practice, the hut has none of the tortoise’s inherently sealed structure isolating the animal inside the shell. Drafty and unstable, huts contain other creatures inside the building materials, which are generally infested with disease carrying insects. Defying the very essence of shelter as an exclusionary agent, rodents and arthropod vectors follow their hosts into the hut, becoming domestic and even changing hosts in the interest of their own survival.

While much of the world has moved from huts to homes with foundations, solid roofs, and walls made with bricks or stones, many still share the tortoise’s compact quarters, albeit without the expert insulation. The more than 1.4 billion people who live in extreme poverty, unable to upgrade their habitations, remain vulnerable to the ecologic realities of tropical and subtropical diseases, some of them woefully neglected―dengue, visceral leishmaniasis, lymphatic filariasis. Also Chagas disease, whose agent *Trypanosoma cruzi* is transmitted by triatomine bugs living in the mud walls of far too many homes in Latin America―not to mention the ever-present scourge of malaria with its ubiquitous and very adaptable vector mosquitoes.

In the southwestern United States, rural environment and housing that did not exclude rodents have been associated with the spread of a novel (now 18 years old) hantavirus. In Argentina, with increased urbanization and encroachment on the subtropical ecosystems of the Paraná Delta, recognized cases of emerging *Rickettsia parkeri* rickettsiosis are likely to escalate. In the Caribbean region of Colombia, as rural populations increase, the potential for arenaviral diseases, whose agents are mainly hosted by rodents, could become a public health concern. In Mongolia, where plague is endemic, the most common source of human infection is contact with and consumption of marmots. In Africa, plague caused by *Yersinia pestis* is transmitted when infected fleas move from feral rodents species to those living in homes. Tick-borne relapsing fever, an infection endemic to many parts of the world, especially Africa and the Mediterranean basin, is mainly attributed to *Borrelia persica* and transmitted by *Ornithodoros tholozani* ticks. These vectors live in caves, soil, and wall crevices, as well as in homes and cow sheds.

The collective and communal nature of the hut’s origins, the coming together of humans to build shelters, may eventually bring about an improved version of this abode. Not only in the interest of less restrictive and more functional dwellings for all but also as part of public health policy. Because the hut is intrinsically connected with the environment (as early builders knew and Kambi so effectively has shown in his painting), persistent and emerging infections transcend its porous walls and reach across the world. Therefore, public health efforts may one day turn the dilapidated and inadequate shelter into a tighter model, with screens, moats, and better insulation, more akin to the tortoise of the initial plan.

**Figure:**
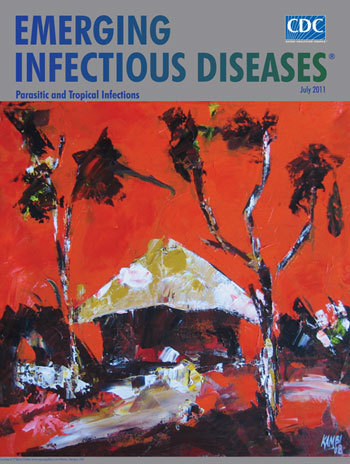
Salum Kambi (b. 1970). *The Village Hut* (2008) Acrylic on canvas (60.32 cm × 60.32 cm). Courtesy of U*Space Gallery (www.uspacegallery.com), Atlanta, Georgia, USA
